# Calcium and Magnesium for Oxaliplatin-Induced Neurotoxicity: Issues in Study Design, Measurement, and Analysis

**Published:** 2015-05-01

**Authors:** Constance Visovsky

**Affiliations:** University of South Florida College of Nursing, Tampa, Florida

Neurotoxicity associated with chemotherapy continues to be of great concern to both advanced practitioners in oncology and the patients they care for. Chemotherapy-induced peripheral neuropathy (CIPN) is considered the dose-limiting toxicity for oxaliplatin, an agent commonly used to treat colorectal cancer (Loprinzi et al., 2014). When administered, oxaliplatin accumulates in the dorsal root ganglia, resulting in axonal neuronopathy (Padman et al., 2014). The neurotoxicity associated with oxaliplatin can be acute or chronic (Tofthagen, McAllister, & McMillan, 2011; Beijers, Jongen, & Vreugdenhil, 2012). Acute neuropathy occurs in 85% to 95% of treated patients, and is manifested by sensitivity to cold, paresthesia, dysesthesia, and muscle contractions of the hands, feet, and circumoral region of the face, while chronic neurotoxicity has a gradual onset with mostly distal to proximal sensory neuropathic symptoms such as paresthesia, dysesthesia, alterations in gait and balance, and decreased vibratory sensation (Tofthagen, McAllister, & McMillan, 2011). Studies of treatments to prevent CIPN are challenging to conduct and occasionally result in conflicting results and clinical recommendations.

## CLINICAL TRIAL CONUNDRUM

One of the issues that plague oncology clinical trials is determining exactly when new treatments tested are ready for implementation into practice. While randomized clinical trials are considered the gold standard for answering research questions, the results of several similar trials can have divergent recommendations or conclusions that inhibit the translation of research into oncology practice. In the case of calcium and magnesium for the prevention of oxaliplatin-induced peripheral neuropathy, a recent study by Loprinzi et al. (2014) failed to support the use of these agents in preventing CIPN. An examination of prior studies can assist the advanced practitioner in understanding the methodologic research issues that led to prior recommendations in favor of calcium and magnesium infusions for patients receiving oxaliplatin-based chemotherapy regimens.

## METHODOLOGIC FACTORS IN PRIOR STUDIES OF CALCIUM AND MAGNESIUM

**Study Design and Sampling**

One of the first considerations is the design of the study to be conducted. The 2004 study by Gamelin et al. was retrospective in nature. Retrospective studies look back over time to factors that influence the study outcome. This study examined the delivery of three different oxaliplatin-based chemotherapy regimens, with or without calcium and magnesium infusions, in a sample of 161 patients receiving second-line treatment for colorectal cancer. Retrospective study designs are more prone to erroneous results due to potential confounding variables or bias not considered or statistically controlled for. For example, the patients enrolled in the 2004 Gamelin study were described as receiving second-line treatment, but the prior treatment received was not described, nor were the neurotoxic effects of prior treatment accounted for in the end analysis. Other important medical variables that could potentially influence the findings, such as cancer disease stage, were not noted. The use of prior neurotoxic chemotherapy or the presence of preexisting neuropathy from non–cancer-related diseases can alter study results. A mix of study participants with more localized disease as well as metastatic disease can also be the cause of disparate study results.

Prospective studies are designed to follow subjects over time, and usually have fewer potential sources of bias and confounding variables than retrospective studies. The CONcePT study referenced in the Loprinzi et al. (2014) article began as a 2 × 2 prospective, randomized trial. Patients were randomized to receive either calcium plus magnesium (Ca/Mg) or placebo while also randomized to either continuous FOLFOX treatment or FOLFOX plus bevacizumab as a "stop and go" treatment (eight cycles of FOLFOX plus bevacizumab followed by maintenance therapy of fluorouracil and leucovorin plus bevacizumab, with reintroduction of oxaliplatin at a later date). However, due to poor accrual, the trial design was changed to a nonrandomized design after 140 patients were enrolled, in which all patients received Ca/Mg. This decision may have been of clinical benefit for patients, but it was at the cost of the research comparison group, significantly reducing the level of evidence for the treatment tested.

The sample size and criteria for study entry are certainly factors that can impact study results. For example, in the study by Ishibashi, Okada, Miyazaki, Sano, and Ishida (2010), the study consisted of only 33 patients enrolled over a 1-year time frame where the participants had differing entry criteria. The patients entered into this study had both unresectable metastatic disease and had undergone resection for metastases, with differing modified FOLFOX6 protocol lengths, and thus, potentially different neuropathic outcomes of Ca/Mg treatment based upon the small sample size, differing study entry criteria, and lastly, the premature closing of the trial. Similarly, in a study by Chay et al. (2010), results may have also been confounded by a very small sample size (n = 27), difference in the samples (metastatic and adjuvant therapies), potentially prior neurotoxic chemotherapy in the metastatic patients, as well as differences in chemotherapy scheduling and doses.

**Measures of the Outcome**

An additional consideration in prior trials of Ca/Mg is how the outcome—in this case, CIPN—was measured. The selection of a standard instrument provides a means of comparing the same outcomes of different trials based on using the same measurement tool. For the two major studies (N08CB and N04C7) that tested Ca/Mg for the prevention of CIPN, the National Cancer Institute Common Terminology Criteria for Adverse Events (CTCAE) was used as the primary neuropathy measure. A letter to the editor (Atkins, 2014) discussing the two disparate trial results found the absolute benefit of neurotoxicity reduction to be only between 1% and 16%.

While the CTCAE provides a standard measure, the manner in which it is used poses additional issues in research. The criteria for the CTCAE were established as cut points to determine treatment toxicity, not as a measure of the symptom experience. There are no standardized instructions or training manual for how to apply or interpret this scale, thus the method for applying this grading scale varies widely, and the interpretation of the meaning of the scale gradations has been inconsistent, potentially affecting study results (Visovsky, Berger, Kosloski, & Kercher, 2008).

On the other hand, there is a decided lack of consensus among experts in the field concerning which instrument or combination of instruments should be used to measure CIPN as a study endpoint, with consideration of both objective and subjective aspects of the CIPN experience. A recent clinical practice guideline for the prevention and management of CIPN from the American Society of Clinical Oncology identified the need for valid and reliable measures of CIPN that assess the extent and severity of CIPN. Such measures should focus on patient-reported outcomes (PROs) as opposed to clinician assessment (Hershman et al., 2014). Supplementing the CTCAE with well-established and validated instruments such as the European Organisation for Research Treatment of Cancer Quality of Life Questionnaire–Chemotherapy-Induced Peripheral Neuropathy 20 (EORTC QLQ-CIPN 20) not only adds important PROs, but makes comparisons between studies possible, as this instrument does not have the inherent limitations of the CTCAE. In this recent study conducted by Loprinzi et al. (2014), the investigator did utilize both the EORTC QLQ-CIPN 20 as the primary endpoint, plus the CTCAE, thus obtaining both PROs as well as a clinician grading that can be used to make study outcome comparisons between past clinical trials.

In addition, the timing of the measure of CIPN could potentially produce conflicting clinical trial results. For example, in oxaliplatin-induced CIPN, the patient may experience a phenomenon known as "coasting," where neuropathic symptoms actually worsen 2 to 6 months posttherapy, or may go on to partially or completely resolve in 40% to 80% of patients within 6 to 8 months of treatment (Argyriou, Bruna, Marmiroli, & Cavaletti, 2012). So, depending upon when the measure is taken in the course of a clinical trial, different results can be obtained.

**Power, Data Analysis, and Missing Data**

Statistical power in a clinical trial refers to the ability to detect a difference in an outcome made by a treatment, when a difference truly exists. There are several reasons power can affect study results in the trials conducted to ascertain if Ca/Mg infusions can indeed prevent oxaliplatin-induced CIPN. Having excessively small sample sizes, or very unequal sample sizes between the treatment and control groups, can affect study results through the loss of statistical power to be able to detect differences between groups. The study by Loprinzi et al. (2014) provided the power analysis for the primary outcome (grade 2+ chronic neuropathy) and met the number of study patients needed in each arm to obtain valid statistical results. Additionally, the statistical analytic plan is well described and considered for the study primary aim. However, the number and types of exploratory analyses on potential secondary outcomes were not well articulated.

In all clinical trials, the issue of missing data becomes an important consideration that can result in the loss of statistical power and bias study results. The percentage and pattern of missing data are also important for the researcher and study statistician to consider. Missing data can take the form of a missing question or missing one or more items on a multilevel instrument or measure (Fox-Wasylyshn & El Masri, 2005). In the Loprinzi study (2014), the missing data are duly reported, as were the statistical considerations for imputation of missing data in the final analysis. When the pattern of missing data is reviewed, it appears that there is a very small (.03–.06) amount of missing data for the baseline sensory score and patients with only one cycle of sensory data. However, data regarding FOLFOX dose reduction and discontinuation by treatment arm denote missing data treatment discontinuance in all three treatment arms that ranges from 12% to 14%. While this is not a primary or secondary study outcome of this trial, this example of missing data illustrates the point that if missing data for the primary aim exceed 10%, the conclusions drawn in the final analysis may be suspect (Cohen & Cohen, 1983).

## CONCLUSION

In summary, the potential for study design, study sample characteristics, study outcome and measurement, as well as considerations of statistical power and analysis, along with missing data can all influence the outcomes of a clinical trial, and thus result in different findings for the same treatment received.
